# Uncovering patterns of inhaler technique and reliever use: the value of objective, personalized data from a digital inhaler

**DOI:** 10.1038/s41533-024-00382-x

**Published:** 2024-08-20

**Authors:** Mark L. Levy, Janwillem W. H. Kocks, Sinthia Bosnic-Anticevich, Guilherme Safioti, Michael Reich, Michael Depietro, Mario Castro, Nabeel Farooqui, Njira L. Lugogo, Randall Brown, Tanisha Hill, Thomas Li, Henry Chrystyn

**Affiliations:** 1 General Practitioner, London, UK; 2 General Practitioners Research Institute, Groningen, Netherlands; 3 Observational and Pragmatic Research Institute, Midview City, Singapore; 4 Groningen Research Institute Asthma and COPD (GRIAC), University of Groningen, University Medical Center Groningen, Groningen, Netherlands; 5 Department of Pulmonology, University of Groningen, University Medical Center Groningen, Groningen, Netherlands; 6 Woolcock Institute of Medical Research, University of Sydney, Glebe, NSW Australia; 7 Teva Pharmaceuticals Europe B.V., Amsterdam, Netherlands; 8 Teva Pharmaceutical Industries Ltd, Tel Aviv, Israel; 9 Teva Branded Pharmaceutical Products R&D Inc., West Chester, PA USA; 10 Division of Pulmonary, Critical Care and Sleep Medicine, University of Kansas School of Medicine, Kansas City, KS USA; 11 Allergy Partners of Fishers, Indianapolis, IN USA; 12 Division of Pulmonary and Critical Care Medicine, Department of Medicine, University of Michigan, Ann Arbor, MI USA; 13 Inhalation Consultancy Ltd, Leeds, UK; 14 Present Address: Incyte Corporation, Newark, DE USA

**Keywords:** Respiratory tract diseases, Therapeutics

## Abstract

Electronic inhalers provide information about patterns of routine inhaler use. During a 12-week study, 360 asthma patients using albuterol Digihaler generated 53,083 inhaler events that were retrospectively analyzed. A total of 41,528 (78%) of the recorded inhalation events were suitable for flow analysis (having a PIF ≥ 18 L/min and <120 L/min). Median PIF, inhalation volume, inhalation duration, and time to PIF for these events steadily decreased between the first and last 10 days of the study, by 5.1%, 12.6%, 15.9%, and 6.4%, respectively. Continuous short-acting beta_2_-agonist (SABA) overuse, defined as ≥2 SABA inhalations/week throughout the study period, was seen in 29% (*n* = 104) of patients. Of 260 patients with ≥1 instance of acute short-term SABA overuse, 55 (21%) had a confirmed exacerbation. Electronic recording of real-life inhaler use can capture valuable, objective information that could inform disease management and clinical decision-making.

## Introduction

Inhalation therapy is the cornerstone of asthma management, delivering medication to the site of action. However, this treatment is reliant upon patients demonstrating correct inhaler technique to allow for the optimal deposition of the medication to achieve a therapeutic effect^1–5^. Despite acceptable inhaler technique demonstrations in clinical practice or during study visits, patients typically do not maintain correct technique over time^6–8^. Suboptimal inhaler technique can result in impaired drug delivery^9,10^ and poor clinical outcomes^7,11–15^. For the past 40 years, the proportion of patients incorrectly using their inhalers has not changed^16^.

Short-acting beta_2_-agonists (SABAs) are bronchodilators used for as-needed symptom relief. The extent of SABA use can be reflective of disease status and outcomes. High SABA use is common in uncontrolled asthma^17–24^ and associated with increased asthma-related unscheduled care, morbidity, and mortality risk^20,21,23,25–29^. Another challenge in asthma management is poor treatment adherence to controller medications^30,31^.

Traditional inhalers utilizing dose counters do not typically provide information on whether a dose was inhaled and if the inhaler technique was correct^32,33^. Digital inhaler systems do provide objective data, including time of use and, in some cases, quality of inhalations^34^. Such objective data can assist in identifying patients with SABA overuse, suboptimal treatment adherence, and/or poor inhaler technique^35,36^. Digital inhalers also offer other potential advantages, including monitoring other more functional and clinically meaningful aspects of inhaler use, such as peak inspiratory flow (PIF) (related to using a DPI with a fast inhalation) and inhalation volume (related to exhaling before an inhalation then inhaling for as long as possible)^3,5,14,37–39^. This technology can be utilized to facilitate objective clinical decision making and the delivery of personalized care to patients with respiratory disease.

**Fig. 1:**
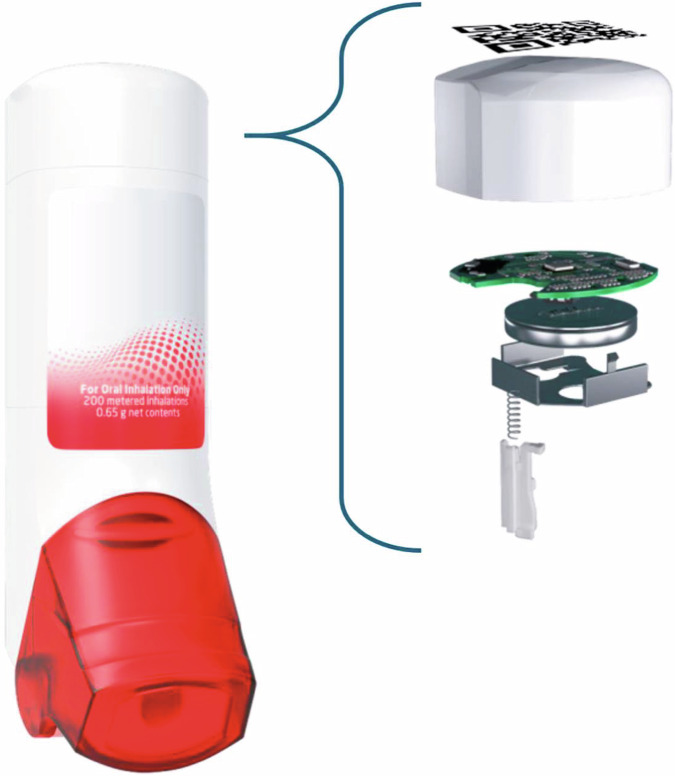
The ProAir^®^ Digihaler^®^ has an integrated electronic module.

The ProAir^®^ Digihaler^®40^ is a Food and Drug Administration (USA)-approved albuterol multidose dry powder inhaler (DPI) with an integrated electronic module (Fig. 1). This Digihaler inhaler objectively measures and records inhalation parameters (PIF, inhalation volume, inhalation duration, and time to PIF), while also providing a time-stamped recording every time the inhaler is used. A recently reported study of asthma patients with a history of exacerbations, using the albuterol Digihaler for 12 weeks, retrospectively analyzed the data downloaded from their inhalers at the end of the study^38^. A machine-learning model predictive of impending exacerbations was developed with features that included increased SABA use and deteriorating inhalation parameters.

The main aim of the current post-hoc analyses of this above-mentioned study^38^ was to explore patterns of inhaled reliever medication use (i.e. episodic increases and overall levels of SABA use) as an objective measure of symptom burden, complemented by real-time data on inhalation parameters in patients using the albuterol Digihaler. We also sought to determine whether information provided by the Digihaler inhaler could be used as a tool to assess inhaler technique.

## Results

### Study population

**Fig. 2:**
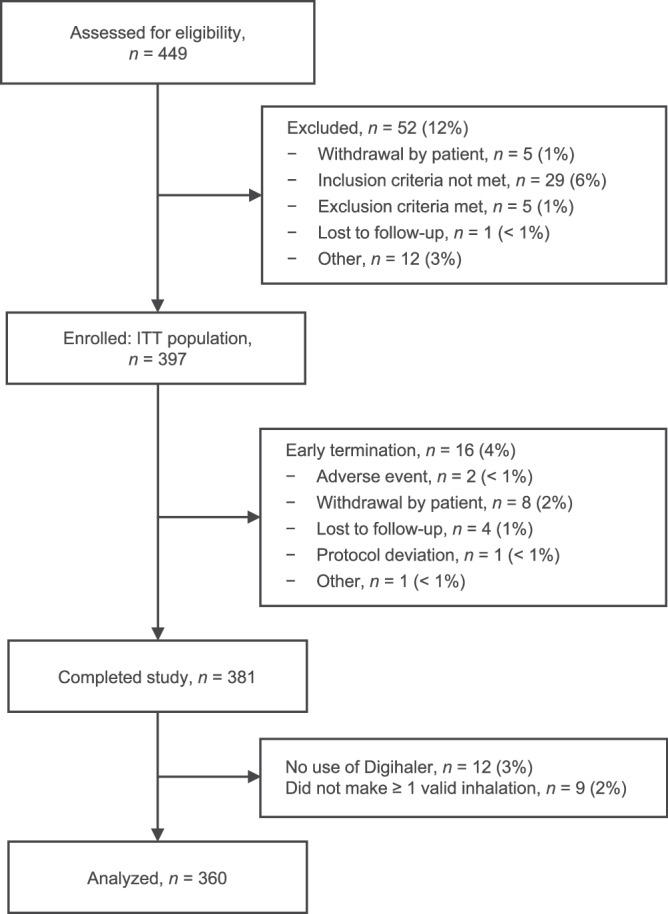
Patient disposition. ITT intention-to-treat.

Overall, 397 patients across 45 investigational centers in the US were enrolled to the intention-to-treat (ITT) population. Of these patients, 381 (96%) completed the study and 360 (91%) made at least one PIF-confirmed inhalation using the albuterol Digihaler (Fig. 2). Baseline demographics and maintenance medication use for the 360 patients who completed the study with at least one PIF-confirmed inhalation using the albuterol Digihaler are shown in Table 1; 80.8% of patients were female and the mean age was 49.9 years.

**Table 1:** Baseline demographics and maintenance medication use.

Demographic	All patients (*N* = 360)	Patients with exacerbations (*n* = 64)	Patients without exacerbations (*n* = 296)
Mean age, years (range)	49.9 (18.0–87.0)	52.0 (25.0–87.0)	49.5 (18.0–82.0)
Females, *n* (%)	291 (80.8)	55 (85.9)	236 (79.7)
Mean body mass index, kg/m^2^ (SD)	33.3 (8.4)	34.1 (7.4)	33.1 (8.6)
Race, *n* (%)			
White	286 (79.4)	50 (78.1)	236 (79.7)
Black or African American	65 (18.1)	14 (21.9)	51 (17.2)
Other	9 (2.5)	0	9 (3.0)
Exacerbations in the 12 months prior to enrollment			
Mean number of exacerbations (SD)	1.5 (1.3)	2.0 (2.3)	1.4 (1.0)
Patients with 1 exacerbation	254 (70.6)	39 (60.9)	215 (72.6)
Patients with 2 exacerbations	69 (19.2)	12 (18.8)	57 (19.3)
Patients with 3 exacerbations	17 (4.7)	4 (6.3)	13 (4.4)
Patients with ≥4 exacerbations	18 (5.0)	9 (14.1)	9 (3.0)
Maintenance medication, *n* (%)			
ICS	17 (4.7)	4 (6.2)	13 (4.4)
ICS-LABA	309 (85.8)	53 (82.8)	256 (86.5)
ICS-LAMA	3 (0.8)	1 (1.6)	2 (0.7)
ICS-LABA-LAMA	29 (8.1)	6 (9.4)	23 (7.8)
Not available	2 (0.6)	0	2 (0.7)

*ICS* inhaled corticosteroid, *LABA* long-acting beta_2_-agonist, *LAMA* long-acting muscarinic agent, *SD* standard deviation.

### Inhalation events

Across the population of patients who completed the study, 53,083 inhaler events were recorded from a total of 736 inhalers from 360 patients. The distribution of PIF across inhalation events is shown in Table 2. A total of 41,528 (78%) of the recorded inhalation events were suitable for flow analysis (having a PIF ≥ 18 L/min and <120 L/min). Their median (interquartile range [IQR]) PIF and inhalation volume were 70.3 (30.1) L/min and 1.18 (0.87) L, respectively. Median (IQR) inhalation duration and time to PIF were 1.33 (0.9) seconds and 0.4 (0.3) seconds, respectively.

**Table 2:** Frequency of inhalation events and PIF by inhalation status.

Inhalation event status	Events, *n* (%)	PIF (L/min), median (IQR)
PIF ≥ 200 L/min	362 (0.7)	227.8 (45.3)
PIF 120– < 200 L/min	3969 (7.5)	135.9 (25)
PIF 45– < 120 L/min	**36,281 (68.3)**	**73.9 (25.9)**
PIF 30– < 45 L/min	**4341 (8.2)**	**39 (6.8)**
Low inhalation flow (PIF 18– < 30 L/min)	**906 (1.7)**	**24.3 (5.4)**
No flow recorded^a^	6635 (12.5)	N/A
Technical errors (such as unexpected multiple inhalations recorded as one event, timeout and unexpected exhalations)	589 (1)	N/A

Inhalation events with a PIF ≥ 18 L/min and <120 L/min (shown in bold type in the table) were included in the analysis.

^a^Inhalation flow below the lower threshold for the MEMS sensor and therefore unable to be recorded – see method section for explanation.

*IQR* interquartile range, *N/A* not applicable, *PIF* peak inspiratory flow.

Of the events that were excluded, 6635 (12.5%) had a PIF below the 18 L/min MEMS threshold to record flow or no inhalation, and 4331 (8.2%) had a PIF ≥ 120 L/min. Technical errors, such as unexpected multiple inhalations recorded as a single event, timeout and unexpected exhalations, accounted for 589 events (1.1%) (Table 2).

**Fig. 3:**
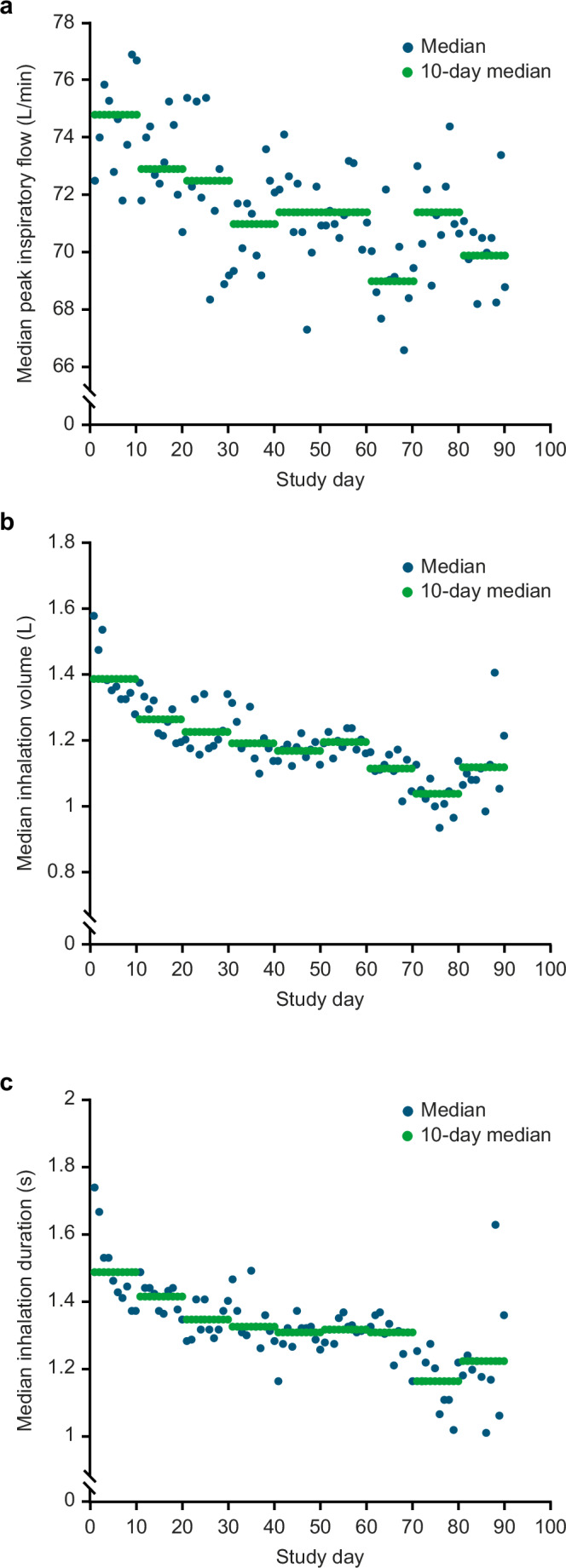
Daily and 10-day medians for inhalation parameters over the full study period. **a** Peak inspiratory flow. **b** Inhalation volume. **c** Inhalation duration. Note: Median calculations exclude inhalation events with PIF <18 L/min or ≥120 L/min. The total number of inhalation events included in the median calculations is 36,246, because patients with study duration <70 days are excluded.

There was a significant difference between the median of the inhalation parameters over the first 10 days and the median over the last 10 days of the study (Table 3). The change in median PIF, inhalation volume, and inhalation duration across the full study period can be seen in Fig. 3. The median percentage change between the first and last 10 days for PIF, inhalation volume, inhalation duration, and time to PIF was −5.1%, −12.6%, −15.9%, and −6.4%, respectively. A typical patient example from this study highlighting these inhalation parameter trends over time is shown in Supplementary Fig. 1.

**Table 3:** Inhalation parameters during the first and last 10 days of the study.

Inhalation parameter	Median (IQR) during the first 10 days of the study	Median (IQR) during the last 10 days of the study	Median difference (IQR)	*p* value
PIF, L/min	77.0 (29.45)	73.1 (25.8)	3.7 (18.25)	0.003
Inhalation volume, L	1.501 (0.904)	1.312 (0.851)	0.242 (0.473)	<0.001
Inhalation duration, seconds	1.58 (0.964)	1.328 (0.943)	0.216 (0.45)	<0.001
Time to PIF, seconds	0.436 (0.346)	0.408 (0.259)	0.026 (0.207)	0.001

Data are shown for patients (*n* = 227) who completed the study and made ≥1 PIF-confirmed inhalation in the first 10 and last 10 days of the study. Medians exclude inhalation events with PIF < 18 L/min or ≥120 L/min. *p*-values were calculated using the Wilcoxon signed-rank test based on differences between patient medians.

*CI* confidence interval, *IQR* interquartile range, *PIF* peak inspiratory flow.

### SABA use

**Fig. 4:**
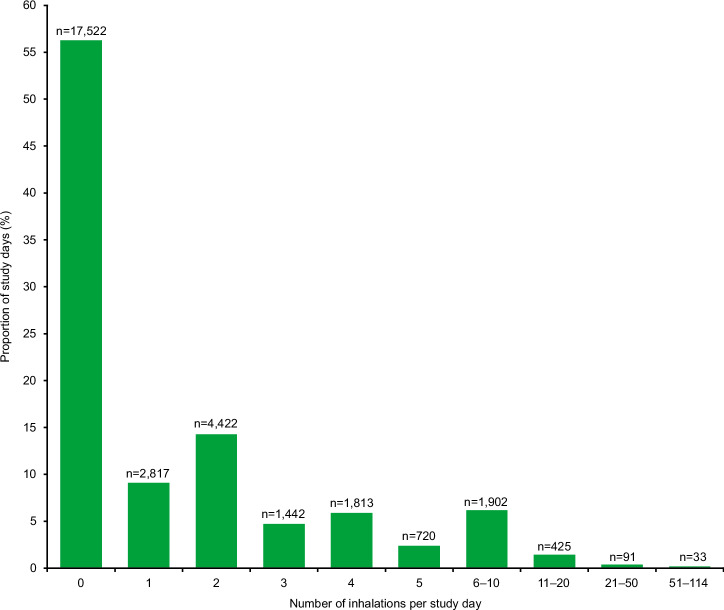
Number of inhalations per day by the proportion of study days. For each category of number of inhalations per study day, proportion of study days (%) was calculated using the formula: (Patient study days for the category/31,187)*100. Displayed n’s reflect numbers of study days; 31,187 is the total number of patient days in the study.

Of the 53,083 inhaler events from patients who completed the study, 36.2% occurred as episodes of single inhalations; the remaining events (63.8%) occurred as episodes of two (44.5%), three (6.5%), four (2.4%), and more than four consecutive inhalations (10.4%) within 60 seconds. The mean number of inhalations per patient per day was 1.70. Most patient days had zero inhalations (56.2%), while 9.0% of patient days had one inhalation, and 14.2% had two inhalations (Fig. 4).

Of the 360 patients who completed the study with at least one PIF-confirmed inhalation using the albuterol Digihaler, 260 (72.2%) patients had a total of 476 bursts of SABA use (Table 4). The mean number of SABA bursts per patient over the 3-month duration of the study was 1.32; 98 (38%) patients had one SABA burst, 111 (43%) had two SABA bursts, 48 (18%) had three SABA bursts, and three (1%) patients had four SABA bursts. Of the 260 patients with SABA bursts, 55 (21%) patients had confirmed American Thoracic Society/European Respiratory Society-defined exacerbations, compared with nine (9%) of the 100 patients who did not have a SABA burst (*Z* = 2.702; two-tailed *p* = 0.007). Of all exacerbations reported during the study (*n* = 74), 40 (54%) were concomitant with a burst of SABA use.

**Table 4:** SABA bursts and continuous overuse.

	Patients, *n* (%)	SABA bursts per patient, mean (SD)	Patients with exacerbations, *n* (%)	Exacerbations, *n*
All patients	360 (100)	1.32 (1.05)	64 (18)	74
No continuous overuse	256 (71.1)	1.02 (0.97)	34 (13)	41
No bursts	96 (37.5)	0.00 (0.0)	9 (9)	9
Bursts	160 (62.5)	1.63 (0.71)	25 (16)	32
Continuous overuse	104 (28.9)	2.07 (0.84)	30 (29)	33
No bursts	4 (3.8)	0.00 (0.0)	0 (0)	0
Bursts	100 (96.2)	2.15 (0.74)	30 (30)	33

SABA bursts were defined as a daily mean of at least three inhalations in the last two days and an increase in daily mean inhalations in the last two days compared with the previous two weeks. Multiple SABA bursts within a 7-day period were counted as one burst. Continuous SABA overuse was defined as at least two inhalations per week, every week, over the study period.

*SABA* short-acting beta2-agonist, *SD* standard deviation.

**Fig. 5:**
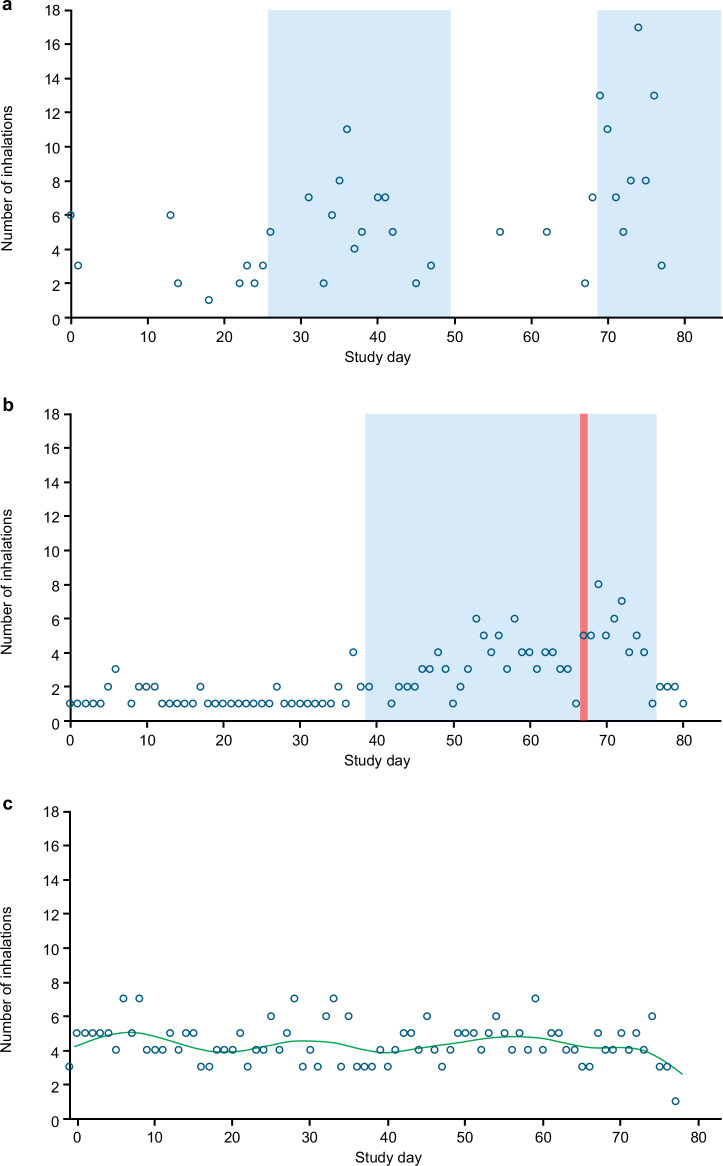
Individual patient examples of SABA use patterns. **a** SABA bursts without a confirmed exacerbation. **b** SABA burst with a confirmed exacerbation. **c** Continuous SABA overuse without SABA bursts or a confirmed exacerbation. Shaded blue areas represent SABA bursts, the red line represents a confirmed exacerbation, and the green line represents trend in SABA use. SABA bursts were defined as a daily mean of at least three inhalations in the last 2 days and an increase in daily mean inhalations in the last 2 days, compared with the previous 2 weeks. Multiple SABA bursts within a 7-day period were counted as one burst. SABA overuse was defined as at least two inhalations per week, every week, over the study period.

**Fig. 6:**
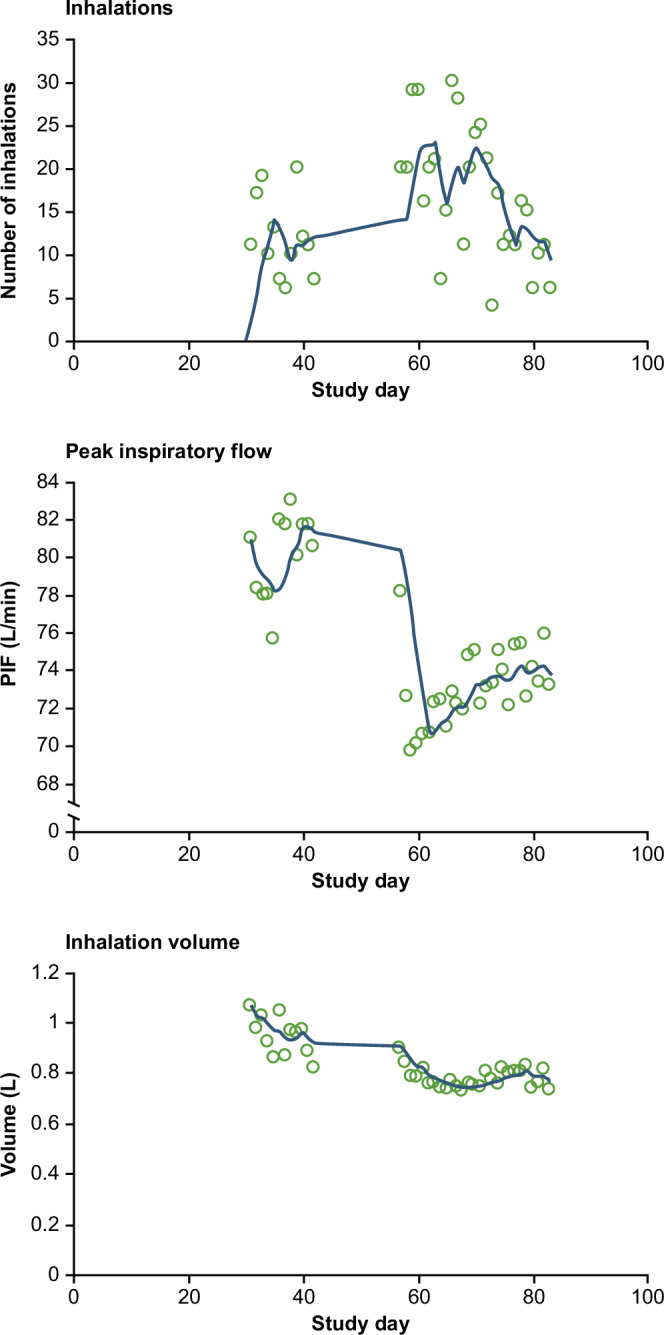
Recorded inhaler usage and inhalation parameters across the study period for an example patient with two periods of SABA overuse. Trendline displays 5-day moving average.

Continuous SABA overuse was seen in 28.9% of patients and a larger proportion of SABA overusers had SABA bursts (96.2%), compared with patients who were not classified as SABA overusers (62.5%) (*Z* = 6.462; two-tailed *p* < 0.001). SABA overusers also had a greater mean number of SABA bursts per patient than SABA non-overusers (2.07 vs. 1.02; Table 4) (*T* = 9.663; two-tailed *p* < 0.001). Individual examples of SABA use patterns are shown in Fig. 5. Figure6 shows a patient example from this study of a patient with a period of SABA overuse and a subsequent period with extremely high SABA use ( ≥ 40 puffs/day). This patient displayed a decrease in inhalation volume between the first and second periods of overuse.

### Safety

Of the 397 patients in the ITT population, 127 (32.0%) experienced any adverse event (AE). Two AEs were deemed by the investigators to be possibly related to treatment; both of these were asthma-related events of moderate severity. Six patients had ≥1 serious AE, none of which were deemed possibly related to treatment. Two patients had ≥1 AE leading to discontinuation, none of which were deemed possibly related to treatment. There were no deaths during the study.

## Discussion

The findings of this study enhance our understanding of the day-to-day burden of asthma, through accurate and objective recording of patterns of real-life SABA use and inhalation parameters. The results demonstrate a high burden of asthma, with continuous SABA overuse and SABA bursts both being frequently observed. While the vast majority of patients maintained sufficient PIF to generate drug dose emission, PIF and inhalation volume were observed to decline from the first 10 days of the study. Reductions in inhalation flow and volume were also observed a few days before reported asthma exacerbations^38^. These findings support the hypothesis that the use of a digitally enhanced SABA inhaler, may provide clinically meaningful data about inhaler technique, level of asthma control and physiological changes during worsening episodes, that could be used to enhance the clinician decision-making process.

This research supports established evidence that subjectively observed patient inhaler technique wanes within the days following single episodes of standard-of-care inhaler training^8,40,41^. However, for the first time, these results show that correct inhaler technique maintenance after training is more complex than just suboptimal PIF. In parallel with the decline in PIF, previously unreported deterioration in both inhalation volume and inhalation duration were also observed. The albuterol Digihaler is a multidose DPI with an integrated electronic module^42^. When using a DPI, the recommended generic technique for the inhalation maneuver is to slowly exhale as far as comfortable (not in the device), inhale as fast as possible from the start of the inhalation and continue inhaling for as long as possible^3^. The CritiKal study^5^ found that errors made during any of these inhalation maneuver steps are clinically important. Inhalation volume ensures that the prepared dose leaves the inhaler and is then delivered to the airway. A reduction in the inhalation volume and the length of the inhalation can be caused by not exhaling before an inhalation or not inhaling for as long as possible. Furthermore, both these are reduced leading up to and during an acute exacerbation^38^. PIF and time to PIF provide the ‘force’ inside the inhaler to de-aggregate the prepared dose into respirable particles with the greatest likelihood to be deposited into the airways. A reduction in these can be caused by not inhaling as fast as possible. Also leading up to and during exacerbations PIF is reduced^38^. Thereby, this research provides further insights on the importance of inhaler technique and its potential links to disease outcomes. The results highlight the importance of counselling patients to exhale first and then to inhale as fast and as long as possible when using their DPI, demonstrated by the highest values of the inhalation parameters directly after training during the enrollment visit.

While a majority of patients were able to achieve a minimum PIF, analysis of PIF values indicates that 12.5% of the inhalations were below 18 L/min. As explained in the methodology section, these inhalations were most likely instances where patients prepared a dose by opening the cap but did not inhale. Other errors were technical issues including multiple inhalations recorded as one event, timeout and unexpected exhalations (1%). Hence, of the attempted inhalations, only 2% had a PIF 18– < 30 L/min while the other 98% were 30 L/min or greater. Dose emission studies have shown consistent albuterol dose emission from the Digihaler inhaler when the PIF is greater than 30 L/min^43^. Despite decline in inhalation parameters throughout the course of the study, the majority of inhalation events were PIF-confirmed inhalations. While the patients in this study did not have access to consistent feedback on inhalation quality via the Digihaler App, it is reasonable to expect that improving clinician and patient access to inhalation parameters data could provide opportunities to address poor inhaler technique.

The Digihaler App, which was not used in this study, enables patients to receive information on the quality of their inhalations. Patients who open the cap but do not inhale would be prompted to make an inhalation, while those patients with a PIF below 30 L/min would be encouraged to try to inhale faster. When the inhalation is 120 L/min or greater, the user could be alerted to be careful about their lips or fingers being too close to the air inlet vent. The Digihaler App defines and presents inhalation data based on PIF, i.e. “good inhalation” (PIF ≥ 45– < 200 L/min), “fair inhalation” (PIF 30– < 45 L/min), “low or no inhalation” (PIF < 30 or no inhalation recorded within 1 minute of the cap being opened), “exhalation” (exhaling/breathing out into the Digihaler inhaler), and “air-vent block” (PIF ≥ 200 L/min). In a study of the Digihaler System (DS), including App and dashboard, inhalation technique based on PIF was maintained throughout the duration of the study, suggesting the effectiveness of technique feedback and monitoring^44^. Those with a history of a gradual reduction in their inhaled volume could be encouraged to exhale before they inhale and continue inhaling for as long as possible.

The results described here illustrate how future versions of the App could support enhanced patient management. Acute increases in SABA use, reductions in PIF and/or reductions in inhaled volume relative to the patient’s typical values for these parameters could prompt reports or alerts to patients and HCPs. These changes have previously been reported to predict the onset of an acute exacerbation^38^. Similarly, the information could be used to improve inhaler technique; for example, if there is a reduction in inhaled volume (which will be linked to a decrease in the inhalation length), the patient could be advised to ensure that they exhale before their inhalation and to inhale for as long as they can. In this regard, it bears highlighting that inhalation parameter values recorded by the Digihaler inhaler are not numerically comparable with values for the analagous spirometric parameters. Notably, the median inhaled volume measurements reported here are markedly lower compared with previously reported forced vital capacity measurements obtained via spirometry in patients with asthma. This is consistent with the findings of a previous validation study: among a group of adults with asthma whose mean FVC was 2.82 L, the mean inhaled volumes measured by an Inhalation Profile Recorder and by the Digihaler were 2.10 L and 1.96 L, respectively^45^. This can be explained in part by the greater internal resistance of the DPI relative to the spirometer—the Digihaler inhaler, having a moderately high internal resistance, does not require a high inspiratory flow for adequate dose delivery; as such, inhaled volumes when using the inhaler tend to be low relative to expiratory parameters measured by spirometry^46,47^. Moreover, patients typically use maximal effort when performing spirometry or using an inhaler in controlled and supervised conditions that contrast with the unsupervised routine use of the Digihaler inhaler in this study. It is therefore not unexpected that the real-world inhaled volumes reported here are lower than those recorded by Chrystyn and colleagues^45^. Clinicians should take this into account when interpreting inhalation parameter data from the DS.

This research has also identified potentially new indicators of poorly controlled asthma and/or disease ‘flare-ups’ through digital capture in real time of acute increases in SABA use, or ‘SABA bursts’. It is established that SABA overuse is associated with increased risks of exacerbation and mortality^20,21,25,26,28,29^, and the increasing trend in SABA overuse^48^ is worrisome as this medication does not target the underlying inflammatory pathology that leads to the asthma symptoms and exacerbations^49,50^. In addition to inhaler technique, this study demonstrated a new perspective on the prevalence of SABA overuse and overreliance. Over the course of this study, those patients who continuously overused SABA were more likely to have exacerbations as well as SABA bursts than patients with lower overall SABA use. The Digihaler System allows for a more reliable way to assess control, based on the objective measure of real-life SABA use instead of a patient’s recollection. There were some patterns of SABA bursts seen in this study that appeared to be similar to patterns associated with exacerbations but did not coincide with a reported exacerbation. These episodes are probably undiagnosed exacerbations as, in the real world, patients forget or do not report them, and HCPs miss the opportunity to diagnose such exacerbations. In this respect, it is interesting to note that all of the patients who had exacerbations and overused SABA during the study recorded at least one SABA burst. These exacerbation-like events are indicative of clinical deterioration or undiagnosed exacerbations, and are probably clinically significant. Further studies are needed to fully understand the implications of such SABA use patterns and to evaluate behavioral systems designed to reduce SABA overreliance and increase use of preventer medication.

These analyses have several limitations. Firstly, because patients in this study did not have access to the Digihaler App, the inhalation event timestamps were not collected in real time but downloaded from the inhaler at the end of the study. In some instances, this data collection occurred several weeks after study completion, which could have resulted in minor inaccuracies in the time of inhalation recorded by the inhaler. Additionally, exacerbations were identified in retrospect via monthly telephone follow-ups by the clinical sites, and not captured in real time. Therefore, some of the exacerbation events may have been subject to recall bias. Furthermore, the definition of continuous SABA overuse excludes patients who used their inhaler continuously for periods of a week or more at a time but did not meet the criteria for an overuser (which stipulated use during every week of the study). In addition, opportunities to provide timely feedback to patients may have been missed owing to the App not being used in this study. This may have contributed to the technique waning effect described above, as patients with suboptimal technique were not alerted to seek medical assistance which might have prevented exacerbations or flare ups.

Although nebulizers were prohibited outside of an exacerbation, use was not recorded and there was no way to prevent patients from using nebulizers during periods of worsening disease. This methodology possibly resulted in periods where albuterol Digihaler use was underestimated around the time of an exacerbation for some patients. Nine (9%) patients in this study had exacerbations without evidence of SABA bursts. This finding illustrates that additional recording and assessment, such as lung function, symptom questionnaires or acoustic monitoring, may be helpful in identifying patients at risk of an exacerbation^51^.

In conclusion, the albuterol Digihaler captures objective information about inhalation events, inhaler parameters, and patterns of SABA use and could be used as a tool to assess inhaler technique. This technology has the potential to ‘profile’ different phenotypes of asthma patients using a combination of inhaler use patterns and inhalation parameters. If these data were routinely available, they could support earlier detection of inhaler technique errors and decline, as well as early warning signs to predict exacerbations. These data could facilitate informed self-management and disease management by clinicians through improved coaching of inhalation technique and clinical decision-making based on objective data. Analysis of these patterns may provide additional information and learnings about long-term asthma outcomes, especially in patients who have fewer documented exacerbations. Understanding this behavior may also help identify markers of uncontrolled disease, such as frequent reliever medication use, highlighting patients who are at risk for poor outcomes.

## Methods

### Study design and participants

This open-label study consisted of a 2-week screening period followed by a 12-week data capture period across 45 study centers in the United States (US) between February 2017 and February 2018 (NCT02969408). Patients included in the study were adults with a physician diagnosis of asthma, which was poorly controlled as defined by an Asthma Control Questionnaire-5 score of ≥1.5^52^, and at least one severe asthma exacerbation during the 12 months prior to screening. Eligible patients were required to be using moderate or high doses of inhaled corticosteroids, equivalent to at least 440 µg daily of fluticasone propionate, with or without other asthma controller medications (long-acting beta_2_-agonist, leukotriene antagonist, long-acting antimuscarinic agent, biologic, or maintenance oral corticosteroids). Patients with any confounding underlying lung disorders other than asthma or who had used any investigational drugs within five half-lives of discontinuation were excluded.

During the baseline visit on study Day 1, patients were trained on correct inhaler technique and, following demonstration of competency, received seven albuterol Digihalers (considered sufficient for the study duration; albuterol 90 μg/dose; up to 1–2 inhalations every 4 hours) for use, as needed, alongside their usual maintenance therapy. For this study, exacerbations were defined based on the 2009 American Thoracic Society/European Respiratory Society recommendations^53^. Moderate exacerbations were defined as worsening asthma that required administration of systemic corticosteroids (SCS) above baseline for at least 3 days, or an unscheduled healthcare provider visit (e.g. doctor’s office or emergency care) associated with an increase in asthma therapy. Severe exacerbations needed both an unscheduled healthcare provider visit and administration of SCS as above.

Patients were required to discontinue all other reliever therapy containing SABA or short-acting antimuscarinic agents and replace them with the albuterol Digihaler as their only reliever inhaler for the duration of the study. Use of nebulized albuterol for treatment of acute exacerbations at home or at the hospital was permitted if deemed necessary by the patient or their physician; this was not recorded. Patients could also continue use of other medications, with any modifications at the discretion of their treating physician. Patients did not have use of the mobile phone software application (App) connected to the Digihaler inhaler, as this was not approved for patient use at the time the study was conducted. The data were downloaded from the Digihaler inhalers at the end of the study; therefore, any patient treatment modifications during the study were based on reports from the patients and not on the data from the albuterol Digihaler. Patients were contacted monthly by phone for collection of information regarding exacerbations (including date of occurrence), maintenance medication, and adverse events.

### Analysis of inhalation parameters

A microelectromechanical system (MEMS) sensor in an integrated electronic module located within the top of the Digihaler inhaler detects changes in pressure generated by inhalation airflow at the mouthpiece. These changes were recorded by the Digihaler inhaler and inhalation parameters (PIF, inhalation volume, inhalation duration, and time to PIF) were calculated^45^. The electronic module also recorded a timestamp upon each use (date and time of inhalation). Each recorded timestamp represented one inhaler use event, i.e. an inhalation.

The MEMS sensor within the Digihaler inhaler has a lower threshold of 18 L/min, below which it cannot discriminate changes in pressure from ambient pressure fluctuations. When this occurs, the Digihaler records a PIF of zero. Previous studies using an Inhalation Profile Recorder to measure PIF (accurate below 5 L/min) involving a total of 300 patients^45,54^, each making three inhalations using either the Digihaler inhaler or the equivalent non-electronic version of this inhaler, have reported that only 2 inhalations (made by the same 7 year old child) had a PIF value that was below 18 L/min. Therefore, recorded PIF values below 18 L/min would almost entirely be due to the patient opening the cap (to activate the electronic module) and making no attempt to inhale. Furthermore, in the aforementioned studies, no patient inhaled with a PIF greater than 110 L/min. A PIF of 120 L/min or greater when using the Digihaler inhaler was, therefore, due to the patient’s lips partially impeding the airflow through the inlet of the device’s air vent. This action would cause a change in the overall resistance of the inhaler, leading to higher internal pressure changes and unrealistically faster PIF and larger inhaled volume values. Inhalations with a PIF ≥ 120 L/min were, therefore, not included in the analysis of the inhalation parameters.

Continuous SABA overuse was defined as use of at least two SABA inhalations per week every week throughout the study period. For the purposes of this analysis, we differentiated continuous SABA overuse from acute short-term high use, and referred to the latter patterns of use as “SABA bursts”. “SABA bursts” were defined as a daily mean of at least three inhalations in the last 2 days and an increase in daily mean inhalations in the last 2 days compared with the previous 2 weeks. Multiple SABA bursts within a 7-day period were counted as one burst.

### Statistical analysis

For the analysis of the inhalation parameters over the 12-week period, those with PIF not detected (less than 18 L/min) or with PIF 120 L/min or greater were excluded, as these were considered to represent patients opening and closing the cap, and possible air vent impediment due to accidental covering of the air vent by the patient, respectively (as previously described above in the methods). Analysis of the PIF measurements revealed a non-normal distribution (Kolmogorov-Smirnov test with *p* < 0.0001). Inhalation parameters for each patient were aggregated into 10-day periods to account for natural intra-individual fluctuations between the inhalations. Descriptive statistics were used to report demographics and SABA use, but no statistical comparisons were made. Statistical analyses were performed using R Statistical Software (version 3.6.1; R Foundation for Statistical Computing, Vienna, Austria).

### Safety

Adverse events were recorded during the study in line with the study protocol, from baseline up to Week 12.

### Reporting summary

Further information on research design is available in the Nature Research Reporting Summary linked to this article.

### Supplementary information

Supplementary Figure 1

Reporting Summary

## Data Availability

The data sets used and/or analyzed for the study described in this manuscript are available upon reasonable request. Qualified researchers may request access to patient level data and related study documents including the study protocol and the statistical analysis plan. Patient level data will be de-identified and study documents will be redacted to protect the privacy of trial participants and to protect commercially confidential information. Please visit www.clinicalstudydatarequest.com to make your request.
