# Supplementary material for: Uncovering patterns of inhaler technique and reliever use: the value of objective, personalized data from a digital inhaler
Source: NPJ Prim Care Respir Med. 2024 Aug 20;34:23. doi: 10.1038/s41533-024-00382-x (PMC11336086; doi:10.1038/s41533-024-00382-x)
Supplement: Supplementary file 1 — Supplementary Figure 1 [file 41533_2024_382_MOESM1_ESM.pdf]

SUPPLEMENTARY FIG. 1. Recorded inhaler usage and inhalation parameters across the study period for an example patient with ‘stable’ inhaler use (2 doses/day, most days)

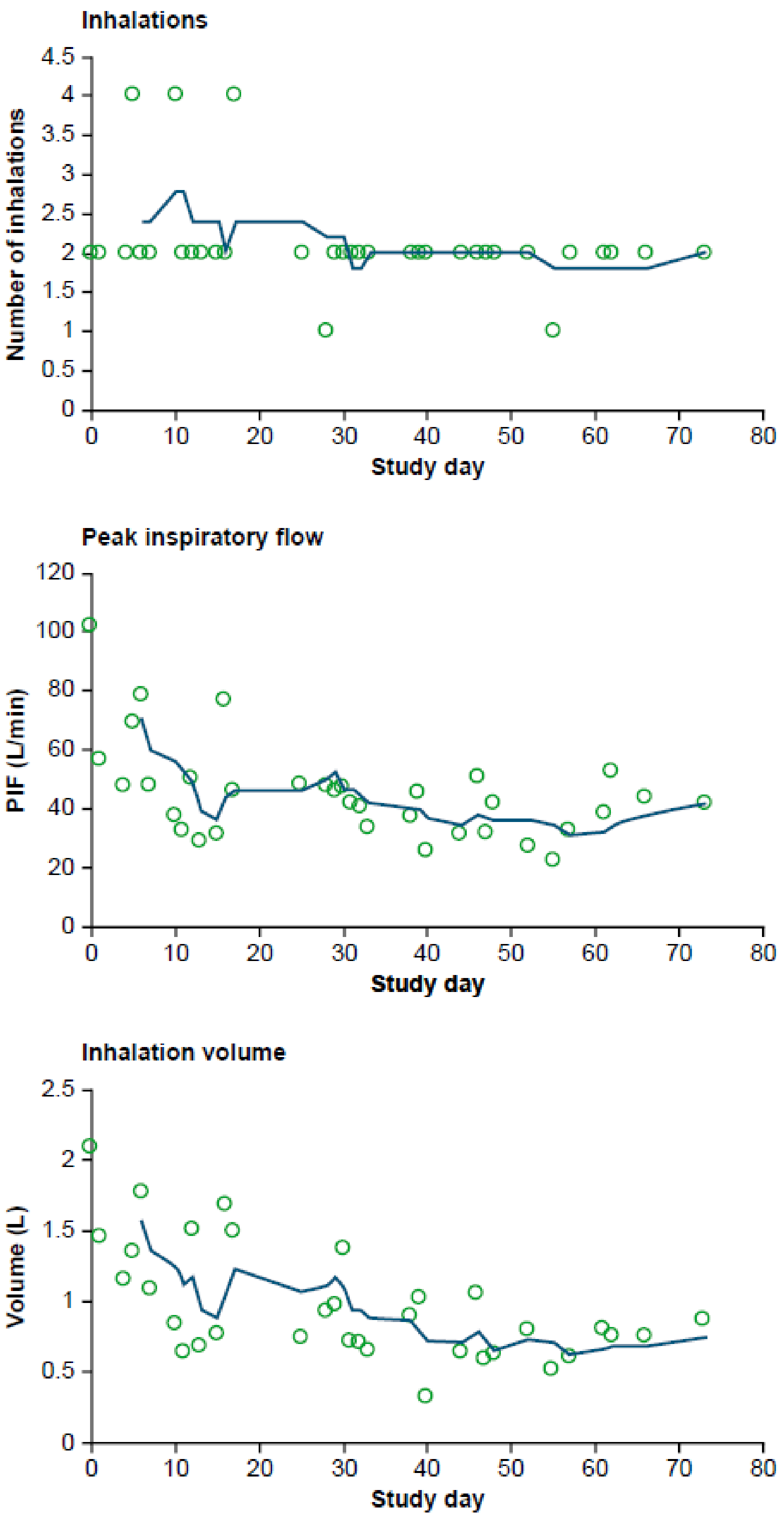

Note: Trendline displays 5-day moving average.
