# Supplementary material for: Uncovering patterns of inhaler technique and reliever use: the value of objective, personalized data from a digital inhaler
Source: NPJ Prim Care Respir Med. 2024 Aug 20;34:23. doi: 10.1038/s41533-024-00382-x (PMC11336086; doi:10.1038/s41533-024-00382-x)
Supplement: Supplementary file 2 — Reporting Summary [file 41533_2024_382_MOESM2_ESM.pdf]

nature portfolio

Corresponding author(s): Mark Levy

Last updated by author(s): Jul 24, 2024

Reporting Summary

Nature Portfolio wishes to improve the reproducibility of the work that we publish. This form provides structure for consistency and transparency in reporting. For further information on Nature Portfolio policies, see our Editorial Policies and the Editorial Policy Checklist.

Statistics

For all statistical analyses, confirm that the following items are present in the figure legend, table legend, main text, or Methods section.

n/a	Confirmed	
☐	☒	The exact sample size (*n*) for each experimental group/condition, given as a discrete number and unit of measurement
☐	☒	A statement on whether measurements were taken from distinct samples or whether the same sample was measured repeatedly
☐	☒	The statistical test(s) used AND whether they are one- or two-sided<br>*Only common tests should be described solely by name; describe more complex techniques in the Methods section.*
☒	☐	A description of all covariates tested
☐	☒	A description of any assumptions or corrections, such as tests of normality and adjustment for multiple comparisons
☐	☒	A full description of the statistical parameters including central tendency (e.g. means) or other basic estimates (e.g. regression coefficient) AND variation (e.g. standard deviation) or associated estimates of uncertainty (e.g. confidence intervals)
☐	☒	For null hypothesis testing, the test statistic (e.g. *F*, *t*, *r*) with confidence intervals, effect sizes, degrees of freedom and *P* value noted<br>*Give P values as exact values whenever suitable.*
☒	☐	For Bayesian analysis, information on the choice of priors and Markov chain Monte Carlo settings
☒	☐	For hierarchical and complex designs, identification of the appropriate level for tests and full reporting of outcomes
☒	☐	Estimates of effect sizes (e.g. Cohen's *d*, Pearson's *r*), indicating how they were calculated

Our web collection on statistics for biologists contains articles on many of the points above.

Software and code

Policy information about availability of computer code

Data collection	ProAir Digihaler (https://www.accessdata.fda.gov/drugsatfda_docs/label/2019/205636Orig1s012lbl.pdf)
Data analysis	R Statistical Software (version 3.6.1)

For manuscripts utilizing custom algorithms or software that are central to the research but not yet described in published literature, software must be made available to editors and reviewers. We strongly encourage code deposition in a community repository (e.g. GitHub). See the Nature Portfolio guidelines for submitting code & software for further information.

Data

Policy information about availability of data

All manuscripts must include a data availability statement. This statement should provide the following information, where applicable:

- Accession codes, unique identifiers, or web links for publicly available datasets
- A description of any restrictions on data availability
- For clinical datasets or third party data, please ensure that the statement adheres to our policy

The data sets used and/or analyzed for the study described in this manuscript are available upon reasonable request. Qualified researchers may request access to patient level data and related study documents including the study protocol and the statistical analysis plan. Patient level data will be de-identified and study documents will be redacted to protect the privacy of trial participants and to protect commercially confidential information. Please visit www.clinicalstudydatarequest.com to make your request.

Research involving human participants, their data, or biological material

Policy information about studies with human participants or human data. See also policy information about sex, gender (identity/presentation), and sexual orientation and race, ethnicity and racism.

Reporting on sex and gender	Data on participant sex are reported in baseline demographics table (Table 1); no sex- or gender-based analyses were conducted as this was not applicable for this descriptive analysis of inhalation parameters.
Reporting on race, ethnicity, or other socially relevant groupings	Data on participant race are reported in baseline demographics table (Table 1); these data (along with other demographic parameters) were obtained from the participants at screening. No race-based analyses were conducted.
Population characteristics	Relevant population characteristics are reported in the baseline demographics table.
Recruitment	Adults with asthma fulfilling study eligibility criteria were recruited from 45 centers across the USA between February 2017 and February 2018. No sources of bias in recruitment were identified.
Ethics oversight	Institutional Review Board IRB #201606556

Note that full information on the approval of the study protocol must also be provided in the manuscript.

Field-specific reporting

Please select the one below that is the best fit for your research. If you are not sure, read the appropriate sections before making your selection.

☒ Life sciences ☐ Behavioural & social sciences ☐ Ecological, evolutionary & environmental sciences

For a reference copy of the document with all sections, see nature.com/documents/nr-reporting-summary-flat.pdf

Life sciences study design

All studies must disclose on these points even when the disclosure is negative.

Sample size	Determination of the sample size is determined in a previous publication: Lugogo NL, DePietro M, Reich M, et al. A Predictive Machine Learning Tool for Asthma Exacerbations: Results from a 12-Week, Open-Label Study Using an Electronic Multi-Dose Dry Powder Inhaler with Integrated Sensors. J Asthma Allergy. 2022;15:1623-1637. Published 2022 Nov 11. doi:10.2147/JAA.S377631
Data exclusions	Exclusions and reasons for exclusion are shown in Figure 2 (Patient disposition).
Replication	Not relevant as this is a descriptive and retrospective analysis.
Randomization	Not relevant as this was not a randomised study; all participants used albuterol Digihaler.
Blinding	Not relevant as this was an open-label study.

Reporting for specific materials, systems and methods

We require information from authors about some types of materials, experimental systems and methods used in many studies. Here, indicate whether each material, system or method listed is relevant to your study. If you are not sure if a list item applies to your research, read the appropriate section before selecting a response.

Materials & experimental systems

n/a	Involved in the study	
☒	☐	Antibodies
☒	☐	Eukaryotic cell lines
☒	☐	Palaeontology and archaeology
☒	☐	Animals and other organisms
☐	☒	Clinical data
☒	☐	Dual use research of concern
☒	☐	Plants

Methods

n/a	Involved in the study	
☒	☐	ChIP-seq
☒	☐	Flow cytometry
☒	☐	MRI-based neuroimaging

Clinical data

Policy information about clinical studies

All manuscripts should comply with the ICMJE guidelines for publication of clinical research and a completed CONSORT checklist must be included with all submissions.

Clinical trial registration	NCT02969408
Study protocol	Available from clinicaltrials.gov (https://classic.clinicaltrials.gov/ProvidedDocs/08/NCT02969408/Prot_000.pdf)
Data collection	45 study centers in the US; between February 2017 and February 2018
Outcomes	Primary outcome measure was asthma exacerbations; these data have been reported previously (Lugogo NL, DePietro M, Reich M, et al. A Predictive Machine Learning Tool for Asthma Exacerbations: Results from a 12-Week, Open-Label Study Using an Electronic Multi-Dose Dry Powder Inhaler with Integrated Sensors. J Asthma Allergy. 2022;15:1623-1637. Published 2022 Nov 11. doi:10.2147/JAA.S377631)

Plants

Seed stocks	Not applicable
Novel plant genotypes	Not applicable
Authentication	Not applicable
